# X-ray Detectors Based on Ga_2_O_3_ Microwires

**DOI:** 10.3390/ma16134742

**Published:** 2023-06-30

**Authors:** Chongyang Zhang, Wenjie Dou, Xun Yang, Huaping Zang, Yancheng Chen, Wei Fan, Shaoyi Wang, Weimin Zhou, Xuexia Chen, Chongxin Shan

**Affiliations:** 1 Henan Key Laboratory of Diamond Optoelectronic Materials and Devices, Key Laboratory of Materials Physics, Ministry of Education, School of Physics and Microelectronics, Zhengzhou University, Zhengzhou 450052, China; 2 Science and Technology on Plasma Physics Laboratory, Laser Fusion Research Center, China Academy of Engineering Physics, Mianyang 621900, China; 3 School of Materials Science and Engineering, Henan University of Technology, Zhengzhou 450001, China

**Keywords:** Ga_2_O_3_, microwire, detector, solar-blind, X-ray

## Abstract

X-ray detectors have numerous applications in medical imaging, industrial inspection, and crystal structure analysis. Gallium oxide (Ga_2_O_3_) shows potential as a material for high-performance X-ray detectors due to its wide bandgap, relatively high mass attenuation coefficient, and resistance to radiation damage. In this study, we present Sn-doped Ga_2_O_3_ microwire detectors for solar-blind and X-ray detection. The developed detectors exhibit a switching ratio of 1.66 × 10^2^ under X-ray irradiation and can operate stably from room temperature to 623 K, which is one of the highest reported operating temperatures for Ga_2_O_3_ X-ray detectors to date. These findings offer a promising new direction for the design of Ga_2_O_3_-based X-ray detectors.

## 1. Introduction

The ability of X-rays to penetrate different materials and densities has brought numerous applications in medical imaging, industrial flaw detection, security checking, and crystal structure analysis [1,2,3,4,5] However, developing X-ray detectors that are highly sensitive and stable has been a significant challenge. Two types of X-ray detectors have been developed to address this problem: indirect and direct conversion detectors [6]. Indirect X-ray detectors use scintillator materials to convert X-rays into fluorescent signals, which are then converted into electrical signals by charge-coupled devices (CCDs). Although indirect detectors are widely used, they have lower photoelectric conversion efficiency and a slower response time due to the afterglow effect of the scintillator [7]. On the other hand, direct X-ray detectors convert X-ray photons directly into carriers, increasing the efficiency of photon utilization, which makes them more suitable for applications requiring higher sensitivity and response speeds. Moreover, these detectors offer advantages in terms of a higher image resolution, lower noise, and a reduced radiation dose, making them an attractive option for medical applications.

Conventional photomultiplier tubes can detect the fluorescent signals converted from X-rays by scintillators with an ultra-fast response and ultra-high internal gain [8]. However, their bulky and fragile nature and large size limit their use. X-ray detectors based on various semiconductor materials, such as CdZnTe [9], high-purity Ge [10], perovskite [11], and amorphous Se [12], have been developed to replace photomultiplier tubes. However, narrow-bandgap semiconductors have some drawbacks compared to wide-bandgap semiconductors when used in X-ray detectors. One of the main disadvantages is their lower photoelectric gain, which can result in a longer collection time to detect X-rays. Additionally, narrow-bandgap semiconductors tend to have higher electron noise, which can reduce the signal-to-noise ratio of the detector. Furthermore, at high temperatures, narrow-bandgap semiconductors can produce more hot electrons, which can adversely affect the performance of the detector.

Wide-bandgap semiconductor materials are essentially suitable for high-voltage, high-frequency, and high-power semiconductor devices [13,14]. Moreover, X-ray detections based on wide-bandgap semiconductors, including GaN, Ga_2_O_3_, SiC, and diamond, possess the advantages of a high breakdown electric field, radiation resistance, and the ability to withstand high temperatures [15,16,17,18,19]. High-temperature X-ray detectors, which can operate without the need for additional cooling systems, find widespread application in industrial scanning, high-energy physics experiments, and space exploration [20]. Ga_2_O_3_ has garnered considerable interest in the fields of power electronics and solar-blind photodetection due to its exceptional performance [21,22]. However, the presence of oxygen vacancies in Ga_2_O_3_ reduces the response speed of the detector and increases the dark current, as has been demonstrated in previous studies [23,24,25]. Ga_2_O_3_-based photodetectors, corresponding to cut-off wavelengths of 250 nm to 280 nm, are expected to replace bulky and fragile photomultiplier tubes for the detection of weak signals in the solar-blind region [26,27,28]. Moreover, Ga_2_O_3_ is also an ideal choice for high-performance X-ray detectors due to its high mass attenuation coefficient [29,30]. Zhang et al. demonstrated high-sensitivity detection and fast responses in Ga_2_O_3_ thin-film avalanche X-ray detectors, while exploring the avalanche multiplication mechanism in Ga_2_O_3_ Schottky diodes [31]. Chen et al. reported an X-ray detector based on Mg-doped Ga_2_O_3_ single crystal, which exhibited excellent performance at room temperature [32]. Semiconductor nano/microstructures have also garnered widespread attention due to their large surface-to-volume ratio, anisotropic geometry, and high crystalline quality compared to bulk or film materials [33,34,35]. Lu and co-workers developed a high-performance solar-blind photodetector array based on Sn-doped Ga_2_O_3_ microwires (MWs). The Sn-doped Ga_2_O_3_ metal–semiconductor–metal (MSM) photodetector exhibited a low dark current of 10 pA and high responsivity of 2409 A/W, suggesting the possibility of developing high-performance X-ray detectors based on Ga_2_O_3_ MWs [36]. However, to date, no such report can be found.

In this study, we present an X-ray detector based on Sn-doped Ga_2_O_3_ MWs. The device exhibits outstanding performance with high responsivity of 1573 A/W and detectivity of 1.5 × 10^14^ Jones under 254 nm illumination. When exposed to X-ray irradiation, the detector’s switching ratio can reach 1.66 × 10^2^, indicating its excellent capability to detect X-rays. Additionally, the Sn-doped Ga_2_O_3_ MW detector remains stable from room temperature to 623 K, with a switching ratio of 2.08 at 623 K. Our findings suggest that Sn-doped Ga_2_O_3_ MWs are promising for X-ray detection under harsh environmental conditions.

## 2. Materials and Methods

### 2.1. Materials

The Sn-doped Ga_2_O_3_ MWs were synthesized using chemical vapor deposition in a tube furnace. A mixed powder of Ga_2_O_3_, SnO_2_, and carbon powder was placed onto an alumina boat, which was then placed inside the furnace along with a sapphire substrate coated with a gold seed layer. The tube was evacuated using a mechanical pump, and a mixture of argon (200 sccm) and oxygen (5 sccm) was injected into the tube during the growth process. The furnace was heated to 1160 °C and maintained at this temperature for 1 h before being cooled to room temperature.

### 2.2. Device Fabrication

To fabricate the Sn-doped Ga_2_O_3_ MW detector, a Sn-doped Ga_2_O_3_ MW was selected and transferred onto a sapphire substrate using tweezers. Then, the electrode patterns were fabricated on both ends of the Sn-doped Ga_2_O_3_ MW using standard lithography techniques. Next, a 100 nm Ag film was deposited onto the sapphire substrate via magnetron sputtering, employing specific process parameters: Ar pressure of 2 Pa and DC power of 50 W. Finally, the desired devices were obtained using the lift-off technique. The distance between the two Ag electrodes was approximately 150 µm.

### 2.3. Characterization

The crystalline properties of the Sn-doped Ga_2_O_3_ MWs were analyzed using an X-ray diffractometer (Smart Lab, Rigaku, Tokyo, Japan) with the Cu Kα line (1.54 Å) as the radiation source. The Raman spectra were obtained using an SOL Instruments (Confotec MR520) spectrometer with a 532 nm laser as the excitation source. High-resolution transmission electron microscopy (HRTEM) and selected area electron diffraction (SAED) were performed using a JEOL JEM-2100F field emission transmission electron microscope. The photoresponse *I*–*V* curves at 254 nm and X-ray irradiation were recorded separately using a triaxial cable connected to a Keithly-4200 SCS semiconductor analyzer at the probe station, ensuring precise and low-noise measurements. The spectral response of the photodetector was evaluated using a photoresponse test system consisting of a 150 W UV enhanced Xe lamp, monochromator, chopper, and lock-in amplifier. The transient optical response was recorded with an oscilloscope (Tektronix DPO 2024B) under the excitation of a 266 nm pulse (with a laser pulse width of 1 ns) and series resistance of 5 MΩ. The X-ray source utilized during the experiment was a MINI-X2 miniature X-ray tube.

## 3. Results and Discussion

**Figure 1:**
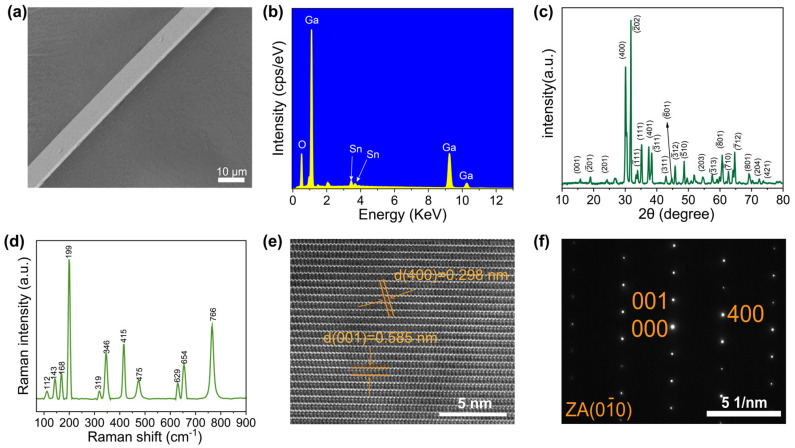
(**a**) SEM image of a single Ga_2_O_3_ MW. (**b**) EDS, (**c**) XRD pattern, and (**d**) Raman spectra of the Ga_2_O_3_ MWs. (**e**) HRTEM image and (**f**) the corresponding SAED pattern of the Ga_2_O_3_ MW.

Figure 1a depicts the scanning electron microscope (SEM) image of an individual Sn-doped Ga_2_O_3_ MW exhibiting a tetra prism structure with dimensions of 8 µm in width and 1 µm in height. Energy-dispersive X-ray spectroscopy (EDS) of the Sn-doped Ga_2_O_3_ MW (Figure 1b) revealed the presence of only Ga, Sn, and O elements, which demonstrated the doping of Sn into Ga_2_O_3_. The elemental content of Ga, Sn, and O was 52.31%, 1.55%, and 46.14%, respectively. The XRD pattern of the Sn-doped Ga_2_O_3_ MWs (Figure 1c) showed distinct diffraction peaks that could be unambiguously attributed to the monoclinic *β*-Ga_2_O_3_ (JCPDS Card No. 00-076-0573). The Raman spectra of the Sn-doped Ga_2_O_3_ MW (Figure 1d) displayed peaks that could all be attributed to *β*-Ga_2_O_3_ [37,38]. The distinctive peak positions and intensities observed in the Raman spectrum aligned precisely with the characteristic vibrational modes of *β*-Ga_2_O_3_. The high-resolution transmission electron microscopy (HRTEM) image of the Sn-doped Ga_2_O_3_ MW (Figure 1e) exhibited clear lattice stripes. The observed lattice stripes corresponded to the (001) and (400) lattice planes of *β*-Ga_2_O_3_, with *d*-spacing of 0.585 nm and 0.298 nm, respectively. The selected area electron diffraction (SAED) pattern (Figure 1f) confirmed that the synthesized Sn-doped Ga_2_O_3_ MW was a uniform single crystal, as evidenced by the reflection angles and the corresponding crystal plane spacing, which could be attributed to the (001) and (400) crystal planes of *β*-Ga_2_O_3_.

**Figure 2:**
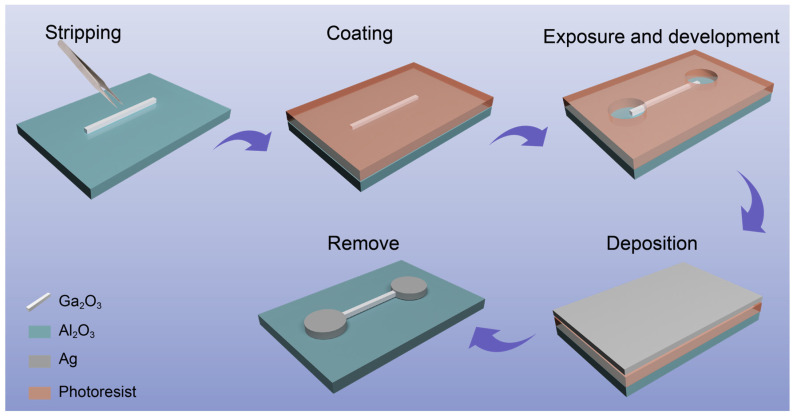
Schematic of the fabrication process of a Sn-doped Ga_2_O_3_ MW detector.

Figure 2 depicts the fabrication steps for a Sn-doped Ga_2_O_3_ MW detector. Firstly, a single Sn-doped Ga_2_O_3_ MW with a line diameter of 8–10 µm and a height of 1 µm is transferred to a sapphire substrate. Next, a photoresist is spin-coated onto the substrate and the Sn-doped Ga_2_O_3_ MW. Electrodes are patterned through photolithography with an electrode pitch of approximately 150 μm. A 100 nm silver film is then deposited on the substrate using magnetron sputtering. Finally, the photoresist is removed via a lift-off process, resulting in the desired device.

**Figure 3:**
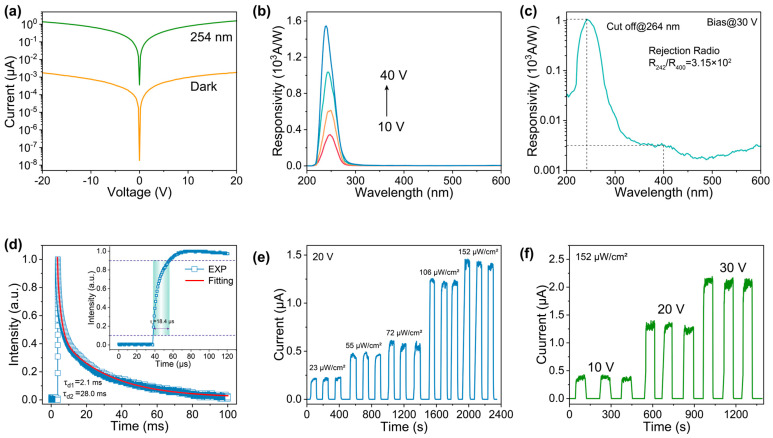
(**a**) *I*–*V* curves of the device in the dark and under 254 nm illumination. (**b**) Response spectra of the device at different biases. (**c**) Response spectrum at 30 V on a semi-log scale. (**d**) Response speed test of the device; the inset shows the amplified rise region. (**e**) Time–dependent response test of the device under different light intensities and (**f**) voltages.

Figure 3a illustrates the current–voltage (*I*–*V*) curves for the Sn-doped Ga_2_O_3_ MW detector. The device can achieve a photo-to-dark current ratio of 10^3^ under the illumination of 254 nm with a light intensity of 106 µW/cm^2^. Figure 3b presents the response spectra of the photodetector from 10 V to 40 V. The data in Figure 3b show that the responsivity can reach up to 1573 A/W at 40 V, indicating the extremely strong photoelectric conversion capability of the photodetector [39,40]. Figure 3c shows the response spectrum of the device at a bias of 30 V on a semi-log scale. The response peak of the Sn-doped Ga_2_O_3_ MW detector is located at 242 nm, while the cut-off wavelength, defined as the wavelength at which the responsivity reduces to 1/e (where e ≈ 2.718) of the maximum responsivity, is approximately 264 nm [41,42]. The UV/Vis rejection ratio, defined as the ratio of the maximum responsivity to the responsivity at 400 nm, is approximately 3.15 × 10^2^.

The detection effect of weak signals can be characterized by the specific detectivity (D*), which can be expressed by the following equation [43]:

(1) $$D*=\frac{R\sqrt{S}}{\sqrt{2eI_{dark}}}$$

Here, *e* is the elementary charge, *S* is the optical power, *I_dark_* is the dark current, and *R* is the responsivity in A/W. For the given values of *R* = 1573 A/W and *I_dark_* = 4.3 × 10^−9^ A at 40 V, the D* of the device is calculated to be 1.5 × 10^14^ Jones. External quantum efficiency (*EQE*), defined as the ratio of the number of collected electron–hole pairs to the number of incident photons, can be determined using the following equation [44]:

(2) $$EQE=\frac{hc}{\lambda e}R\times 10^{2}\%$$

Here, *h*, *c*, and *λ* are the Plank’s constant, speed of light, and optical wavelength, respectively. At a bias of 40 V and under 254 nm illumination of 106 µW/cm^2^, the *EQE* of the Sn-doped Ga_2_O_3_ MW detector is estimated to be 7.9 × 10^5^%.

Figure 3d displays the transient response of the detector, which exhibits a rise time (*τ_r_*, defined as the duration required to increase the photocurrent from 10% to 90% of the maximum value) of 18.4 μs approximately. Moreover, the decay process is well described by a double exponential equation [45]:

(3) $$I(t)=A\exp(-t/\tau_{1})+B\exp(-t/\tau_{2})+I_{0}$$

Here, *A* and *B* are constants, and *τ*_d1_ and *τ*_d2_ indicate the fast and slow decay time constants, respectively. *I*_0_ is the steady-state dark current contribution. The fitting of the decay process to Equation (3) yields *τ*_d1_ = 2.1 ms and *τ*_d2_ = 28 ms.

Figure 3e,f present the time-dependent photocurrent (*I*–*t*) of the device under various light intensities and voltages. The measurements were taken with a periodic on–off cycle of the UV lamp, which was set to switch every 70 s. Upon activating the UV lamp, the current rose sharply to a peak value and then gradually leveled off. Conversely, the current dropped steeply when the UV lamp was turned off. The detector exhibited periodic switching between high- and low-current states, with the maximum photocurrent increasing with higher voltages and light intensities.

**Figure 4:**
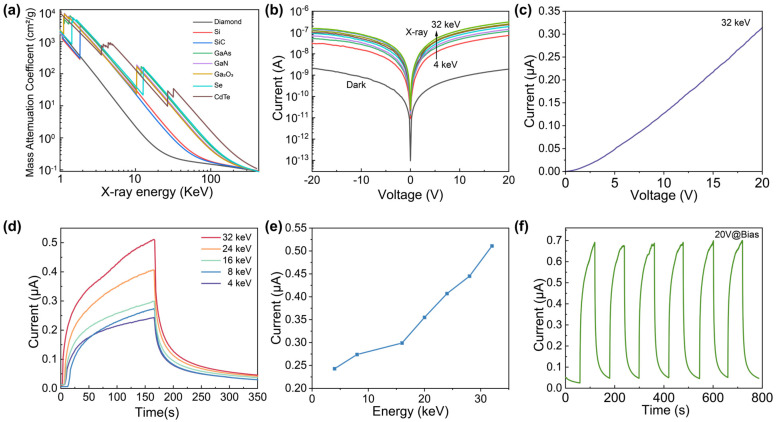
(**a**) Plots of linear attenuation coefficients for different materials. (**b**) *I*–*V* curves of the detector under different X-ray energy (From 4 keV to 32 keV in steps of 4 keV). (**c**) *I*–*V* characteristics for the detector under X-ray irradiation of 32 keV. (**d**) *I*–*t* curves of the detector under X-ray irradiation of different energy. (**e**) Maximum current versus X-ray energy. (**f**) Multi-cycle response of the detector under X-ray of 32 keV.

Figure 4a displays the linear attenuation coefficients of various semiconductor X-ray sensing materials [26]. Within the low-energy X-ray range (1–30 keV), the mass attenuation coefficients of Ga_2_O_3_ are comparable to those of CdTe and Se, which are significantly higher than that of Si [46]. Additionally, the *I*–*V* curves of the detector under X-ray irradiation with different energy are shown in Figure 4b. The results demonstrate that the X-ray radiation current is considerably larger than the dark current, with a difference of roughly two orders of magnitude between the light and dark currents. Furthermore, the maximum detector current increases with the X-ray energy, reaching a photocurrent of 3.14 × 10^−7^ A at 20 V bias under the irradiation of 32 keV X-rays. This can be attributed to the internal photoelectric effect. As the X-ray energy increases, more valence band electrons are excited to the conduction band, leading to an increase in photocurrent [31]. Figure 4c shows the *I*–*V* characteristics of the detector under X-ray irradiation of 32 keV, where the current increases linearly with increasing voltage. In Figure 4d, the response of the detector in the range of X-ray energy from 4 keV to 32 keV is presented. As shown in Figure 4e, the maximum current increases with the X-ray energy. Figure 4f displays the repeatable response of the detector at 20 V under 32 keV X-ray energy. The X-ray is turned on and off periodically at 60 s intervals. The current increases rapidly when the X-ray source is turned on and drops sharply when the X-ray source is turned off. Overall, the results suggest that Ga_2_O_3_ MWs are promising for X-ray detection, and the detector exhibits a robust and repeatable response to X-ray irradiation.

**Figure 5:**
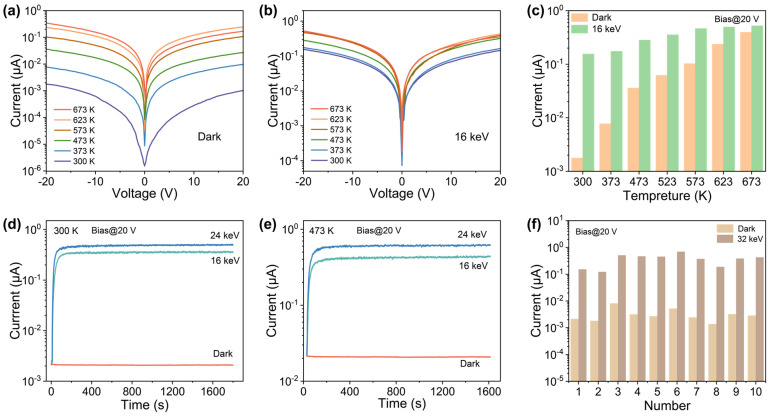
(**a**) *I*–*V* curves for the device at different temperatures in the dark and (**b**) under X-ray irradiation of 16 keV. (**c**) Photocurrent and dark current of the device at different temperatures. Sn-doped Ga_2_O_3_ MW detector stability testing at (**d**) 300 K and (**e**) 473 K. (**f**) Statistics of dark current and photocurrent for 10 X-ray detectors.

Figure 5a shows the dark *I*–*V* curves of the X-ray detector at temperatures ranging from 300 K to 673 K, while Figure 5b illustrates the *I*–*V* curves under 16 keV X-ray irradiation, also across the same temperature range. As the temperature increases, both the dark and photocurrents increase due to more valence band electrons transitioning to the conduction band [47]. Remarkably, even at 623 K, the on–off current ratio remains at 2.08, as illustrated in Figure 5c, indicating that the X-ray detector can operate reliably at high temperatures. Furthermore, the stability of the detector was evaluated by continuously measuring the current under X-ray irradiation at room temperature (300 K) for 1600 s, as shown in Figure 5d. The detector exhibited excellent stability throughout the measurement period, with the excitation current remaining stable across the various operating voltages tested. This result suggests that the device responds reliably and consistently to X-ray irradiation.

Additionally, the performance of the device was evaluated at high temperatures to test its possibility to operate in harsh environments. Figure 5e shows that at 473 K, both the currents in the dark and under X-ray irradiation increased compared to room temperature. However, despite the increased currents, the switching ratio of the device remained at approximately 10, indicating that it can perform well under challenging operating conditions. We evaluated the uniformity of 10 detectors under X-ray and darkness, as shown in Figure 5f. The detectors showed good uniformity under X-ray irradiation of 32 keV, with the light-to-dark current ratio exceeding 100.

As summarized in Table 1, our Sn-doped Ga_2_O_3_ detectors excel in solar-blind and X-ray detection, with exceptional responsiveness and response times, surpassing other Ga_2_O_3_ photodetectors.

## 4. Conclusions

In this study, we present a Sn-doped Ga_2_O_3_ MW detector capable of detecting ultraviolet light and X-rays with exceptional stability and reproducibility, even under varying light intensities, X-ray energies, and biases. Our device exhibits remarkable performance, with high responsivity of 1573 A/W, an EQE of 7.9 × 10^5^%, and detectivity of 1.5 × 10^14^ Jones when exposed to 254 nm illumination of 106 µW/cm^2^. Moreover, when irradiated with X-rays, the device achieves a switching ratio of 166. Notably, it reliably operates over extended temperature ranges, from room temperature to 623 K. These findings present a novel approach to developing Ga_2_O_3_ detectors, which hold great potential for future applications.

## Figures and Tables

**Table 1:** Comparison of the performance of Ga_2_O_3_-based detectors.

Material	I_dark_(A)	R(A/W) @254 nm	Rise/Decay time (s)	Structure	Work Temperature	Reference
GaO_X_	≈10^−13^	66.7	0.08/−@254 nm	MSM	573 K	[22]
Ga_2_O_3_	≈10^−12^	-	<0.3/<0.3@X-ray	MSM	-	[29]
Ga_2_O_3_	≈10^−10^	-	0.132/0.037@X-ray	MSM	-	[31]
Ga_2_O_3_	≈10^−10^	-	<0.2/<0.2@X-ray	MSM	-	[32]
Ga_2_O_3_ MWs	≈10^−11^	2409	0.0079/1.18@254 nm	MSM	-	[36]
Ga_2_O_3_	1.6 × 10^−11^	295	1.7 × 10^−6^/1.1 × 10^−4^@254 nm	MSM	-	[44]
Ga_2_O_3_	≈10^−9^	0.0485	8 × 10^−7^/10^−4^@254 nm	MSM	-	[45]
Ga_2_O_3_/ZnO	2.3 × 10^−8^	1300	<2 × 10^−5^/4.2 × 10^−5^@254 nm	Heterostructure	-	[48]
Ga_2_O_3_	≈10^−12^	-	<0.5/−@X-ray	MSM	-	[49]
Ga_2_O_3_ MWs	≈10^−9^	1573	1.8 × 10^−5^/0.03@254 nm 43.4/22.3@X-ray	MSM	623 K	This work

## Data Availability

Not applicable.
